# hsa_circ_0000520 influences herceptin resistance in gastric cancer cells through PI3K‐Akt signaling pathway

**DOI:** 10.1002/jcla.23449

**Published:** 2020-07-23

**Authors:** Xukun Lv, Peizhe Li, Jinkai Wang, Hengling Gao, Yingrui Hei, Jianxian Zhang, Shuliang Li

**Affiliations:** ^1^ Department of Gastrointestinal Surgery The Second People's Hospital of Liaocheng Linqing China

**Keywords:** drug resistance, gastric cancer, Herceptin, hsa_circ_0000520, PI3K‐Akt

## Abstract

**Background:**

To investigate whether hsa_circ_0000520 affects Herceptin resistance in gastric cancer by regulating the PI3K‐AKT signaling.

**Methods:**

The expression of hsa_circ_0000520 was detected by qRT‐PCR in gastric cancer tissues and cell lines. A Herceptin‐resistant gastric cancer cell was established. PcDNA and pcDNA‐hsa_circ_0000520 were transfected into NCI‐N87R cells and treated with Herceptin at a concentration of 10 μg/mL for 24 hours. MTT tested cell proliferation, and apoptosis was measured by flow cytometry. IGF‐1 treatment was used to activate PI3K‐Akt signaling. The expression levels of related proteins were detected.

**Results:**

The expression of hsa_circ_0000520 was reduced in gastric cancer tissues and cell lines, and hsa_circ_0000520 in NCI‐N87R cells was significantly lower than that of NCI‐N87 cells. Compared with the CON group, the cell viability of the Herceptin group was significantly reduced, the apoptosis rate was significantly increased, the level of Bax protein was significantly increased, and the levels of Bcl‐2, p‐PI3K, and p‐Akt protein were significantly reduced. Compared with the Herceptin + pcDNA group, the cell viability of the Herceptin + hsa_circ_0000520 group was significantly reduced, the apoptosis rate was significantly increased, the level of Bax protein was significantly increased, and the levels of p‐PI3K and p‐Akt proteins were significantly reduced. After IGF‐1 treatment, the cell viability was significantly increased, the apoptosis rate was significantly reduced, the level of Bax protein was significantly reduced, and the level of Bcl‐2 protein was significantly increased.

**Conclusion:**

Hsa_circ_0000520 overexpression may reverse the Herceptin resistance of gastric cancer cells by inhibiting the PI3K‐Akt signaling pathway.

## Graphical abstract

Effect of activating PI3K‐Akt signaling pathway on hsa_circ_0000520 overexpression and Herceptin‐treated NCI‐N87R cells. A, MTT was used to determine proliferation of NCI‐N87R cells. B, Apoptosis of Herceptin‐treated NCI‐N87R cells was detected by flow cytometry. C, Apoptosis‐related proteins was tested by Western blot. Note: Compared with Herceptin + hsa_circ_0000520 group, * P <0.05.

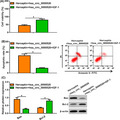

## 1 INTRODUCTION

Gastric cancer is a clinical malignant tumor of digestive system. In recent years, the morbidity and mortality of gastric cancer in China have increased year by year, which has seriously threatened the safety of human life. Most patients were in advanced gastric cancer at the time of diagnosis. The poor prognosis is due to the highly aggressive tumors. At present, surgery and radiotherapy and chemotherapy are mainly used to treat gastric cancer, but the 5‐year survival rate is still very low. Therefore, finding genes contributed to development of gastric cancer is helpful to improve treatment effect of gastric cancer and the prognosis.^1^, ^2^

Circular RNA (circRNA) is a class of RNA molecules with a ring structure formed by 5´ and 3´ ends. It is abnormally expressed in tumors and to be a marker of diagnosis and therapeutic target. It has the characteristics of stability, tissue specificity and high conservation, and may become molecular marker for early diagnosis in gastric cancer. As the study indicated, circRNA can regulate the gastric cancer development by acting as sponge molecules of microRNA (miRNA).^3^, ^4^, ^5^, ^6^, ^7^

Herceptin is a humanized monoclonal antibody designed by combining non‐specific human IgG stabilization zone with specific mouse anti‐HER2 antigen determination cluster through genetic engineering, which can specifically bind to HER2. And it has been reported that Herceptin acted as an antitumor role in gastric cancer. Although Herceptin has obvious effects, some patients with gastric cancer still have Herceptin resistance.^8^ Therefore, it will be the focus of research that how to improve the treatment effect of gastric cancer, clarify the Herceptin resistance mechanism, and study on reversing Herceptin resistance.

In gastric cancer, the expression level of circular RNA _0000520 (circular RNA _0000520, hsa_circ_0000520) was detected and found to be significantly decreased. Further study found that hsa_circ_0000520 level was closely related to the clinical stage and the symptoms of gastric cancer patients, so it could be used as a clinical marker for gastric cancer.^9^ However, it has not been elucidated the underlying mechanism of hsa_circ_0000520 in gastric cancer development. Studies have shown that activation of the PI3K/AKT signaling can promote the development of gastric cancer.^10^ However, it is unknown whether PI3K‐Akt was involved in hsa_circ_0000520 regulating Herceptin resistance of gastric cancer. Therefore, present study mainly explored the role of hsa_circ_0000520 function on the Herceptin resistance in gastric cancer, and its regulatory effect on the PI3K‐Akt. It provides the experimental evidence to reduce Herceptin resistance of gastric cancer cells.

## 2 MATERIAL AND METHOD

### 2.1 Material and reagent

There were 30 patients with gastric cancer admitted in the Second People's Hospital of Liaocheng from October 2017 to December 2018 to be selected as the research subjects. All patients were confirmed to be gastric cancer by pathology, including 20 males and 10 females, with an average age of 56.35 ± 12.30 years. During the operation, the gastric cancer tissue and its adjacent tissue were removed and recorded as cancer group and normal group, respectively. They were stored in a −80℃ ultra‐low temperature refrigerator.

All patients in our study provided informed consent. This study was approved by the Medical Ethics Committee of the Second People's Hospital of Liaocheng and met the standard set in the Declaration of Helsinki.

Normal gastric mucosal epithelial cells GES‐1 and cancer cells NCI‐N87 were purchased from ATCC; Herceptin was purchased from Shanghai Roche Pharmaceutical Co., Ltd; PI3K‐Akt activator insulin growth factor‐1 (IGF‐1), DMEM, and fetal calf serum were purchased from Gibco, USA; trypsin, Lipofectamine2000, reverse transcription kit, and real‐time PCR kit were purchased from Thermo Fisher Scientific; TRIzol reagent was purchased from Invitrogen, USA; pcDNA3.1 was purchased from Shanghai solarbio Bioscience & Technology Co., LTD; methylthiazolyl tetrazolium (MTT) and dimethylsulfoxide (DMSO) were purchased from Sigma Company of the United States; Annexin V (Isosulfur) FITC/ Propidium Iodide (PI) apoptosis kit was purchased from Shanghai Langzhi Biotechnology Co., Ltd; RIPA lysate was purchased from Nanjing Senberga Biotechnology Co., Ltd; diquinoline formic acid (bicinchoninic acid, BCA) protein quantitative detection kit and enhanced chemiluminescence reagent (electrochemiluminescence (ECL) were purchased from TransGen Biotech Co., Ltd; rabbit anti‐human B lymphoma‐2 related protein (Bcl‐2‐associated X protein, Bax) and B‐cell lymphoma‐2 (Bcl‐2) antibodies were purchased from CST, USA; rabbit anti‐human PI3K, phosphorylated phosphatidylinositol‐3‐Kinase (phospho‐PI3K, p‐PI3K), Akt, and phosphorylated protein kinase B (phospho‐Akt, p‐Akt) antibodies were purchased from Santa Cruz Company, USA; Horseradish peroxidase labeled goat anti‐rabbit IgG was purchased from Abcam, USA. The secondary antibody was purchased from Abcam, USA.

### 2.2 Methods

#### 2.2.1 Experimental grouping

Establishing gastric cancer cells NCI‐N87R of Herceptin‐resistant:

NCI‐N87 cells in logarithmic growth phase were cultured in DMEM complete medium containing Herceptin.

Set the concentration gradient of Herceptin to 2.5 μg/mL, 5 μg/mL, 10 μg/mL, 15 μg/mL, 20 μg/mL. NCI‐N87 cells were finally treated with Herceptin at a concentration of 10 μg/mL for 8 months. It was selected the cell line which could stably proliferate in the presence of Herceptin, and named as NCI‐N87R. We detected cell viability of NCI‐N87R every 15 days during 8 months.

Normal gastric mucosal epithelial cells GES‐1 and gastric cancer cells NCI‐N87 and NCI‐N87R were cultured in DMEM complete medium containing 10% fetal bovine serum, penicillin, and streptomycin (100 μg/mL), and placed them at 37℃ in a 5% CO2 incubator. When the confluence of cell growth reaches 80%, digest with 0.25% trypsin, add DMEM complete medium to prepare a cell suspension, and adjust the cell density to 5 × 10 4 cells/ mL. It was inoculated 96‐well plates at a density of 100 μL per well, treated with Herceptin (10 μg/mL) for 24 hours, and recorded as the Herceptin group. The normal cultured cells were recorded as the CON group. Refer to the instructions of the Lipofectamine 2000 Transfection Reagent, PcDNA and pcDNA‐hsa_circ_0000520 were transfected into NCI‐N87R cells separately and then treated with Herceptin (10 μg/mL) for 24 hours, which were recorded as Herceptin + pcDNA group and Herceptin + hsa_circ_0000520 group. The following experiment was to investigate whether hsa_circ_0000520 could be effective by regulating the PI3K‐Akt signaling pathway. It was transfected pcDNA‐hsa_circ_0000520 into NCI‐N87R cells, respectively, and then treated PI3K‐Akt signaling pathway activator (100 ng/mL) with Herceptin at a concentration of 10 μg/mL Co‐processing for 24 hours, which was recorded as Herceptin + hsa_circ_0000520 + IGF‐1 group.

#### 2.2.2 Quantitative Real‐time PCR (qRT‐PCR)

RNA was isolated from frozen gastric cancer tissues, paracancerous tissues, GES‐1, NCI‐N87, NCI‐N87R and each group of transfected NCI‐N87R cells by TRIzol method, and RNA concentration and purity were detected with Nanodrop2000c ultra‐micro spectrophotometer. The RNA OD 260 / OD 280 is between 1.8‐2.0.

Reverse transcription system: RNA 2 μL, 10 × RT Buffer 2 μL, dNTP 0.4 μL, Multiscripe RT 1 μL, 10 × Random Primer 2 μL, RNase‐Free ddH 2 O to complete the system to 20 μL;

Reaction conditions: 25℃ 10 minutes, 37℃ 60 minutes, 95℃ 5 minutes, 4 stored. Forward primer 5'‐GGTGGTGAGGCAGTTGAGAA‐3 ', reverse primer: 5'‐CCAGCATTTCCATACGCCTC‐3'; GAPDH forward primer 5'‐AACGGATTTGGTCGT ATTG‐3 ', reverse primer 5'‐GGAAGATGGTGATGGGATT‐3', the primers were designed and synthesized by Shengong Bioengineering Co., Ltd.

The qRT‐PCR reaction using cDNA as template, qRT‐PCR reaction system: cDNA 2 μL, Real‐Time Master Mix 10 μL, 1 μL each of forward and reverse primers, RNase‐Free ddH 2 O makes up the system to 20 μL; reaction conditions: 95℃ 2 minutes, 95℃ 15 seconds, 60℃ 1 minutes, 72℃ 30 seconds (cycle 40 times). Circ_0000520 was taken GAPDH as internal parameter and calculate the relative expression by using 2‐ ΔΔCt method.

#### 2.2.3 MTT assay

NCI‐N87R cells in the logarithmic growth phase of each group were inoculated into a 96‐well plate (5 × 10 3 cells/ well). Each group is set up with 3 duplicate wells which is added with 20 μL of MTT reagent individually, cultured for 4 hours, and discarded supernatant.

To add 150 μL DMSO to each well, detect the relative absorbance (OD) of each well at a wavelength of 490 nm by using a microplate reader.

#### 2.2.4 Flow cytometry

NCI‐N87R cells in logarithmic growth phase of each group were digested with 0.25% trypsin, add DMEM medium to prepare cell suspension, wash with pre‐chilled PBS, centrifuge at 12,000 g for 6 minutes at 4℃, and discard the supernatant.

Cells were washed with pre‐chilled PBS, centrifuge again under the same conditions, and discard the supernatant. Add 500 μL binding buffer to resuspend cells. Then, add Annexin VFITC and PI according to the apoptosis kit, and incubate for 10 min at room temperature with shaking. Finally, detect the apoptosis rate of each group by FACS Calibur flow cytometry and Cellauest software.

#### 2.2.5 Western blot

NCI‐N87R cells in logarithmic growth phase of each group were lysed by 400 μL RIPA lysate on ice for 30 minutes and centrifuged at 12,000 g for 6 minutes at 4°C to extract total protein.

The protein concentration was measured by BCA method, which followed the instructions of the BCA Protein Quantification Kit seriously. Add 5 × SDS loading buffer to the protein sample, and boil for 10 min in boiling water to denature the protein.

To isolate the protein, transfer the membrane and block by using sodium lauryl sulfate‐polyacrylamide gel electrophoresis (SDS‐PAGE).

Add PI3K, p‐PI3K, Akt, p‐Akt, Bax, Bcl‐2 and GAPDH primary antibody diluent, and incubated at 4℃ for 24 hours, and washed with TBST.

Add secondary antibody diluent (1:5000), TBST washing, dripping ECL, exposure and development in dark room. Band gray value was calculated by QuantityOne software.

#### 2.2.6 Statistical processing

SPSS 21.0 statistical software was used to analyze the data. The measurement data conformed to the normal distribution. The data were all expressed by （$\bar{x}$±*s*）. The data of each group are statistically significant with *P* < .05.

## 3 RESULTS

### 3.1 hsa_circ_0000520 was decreased in gastric cancer tissues and cells

**FIGURE 1:**
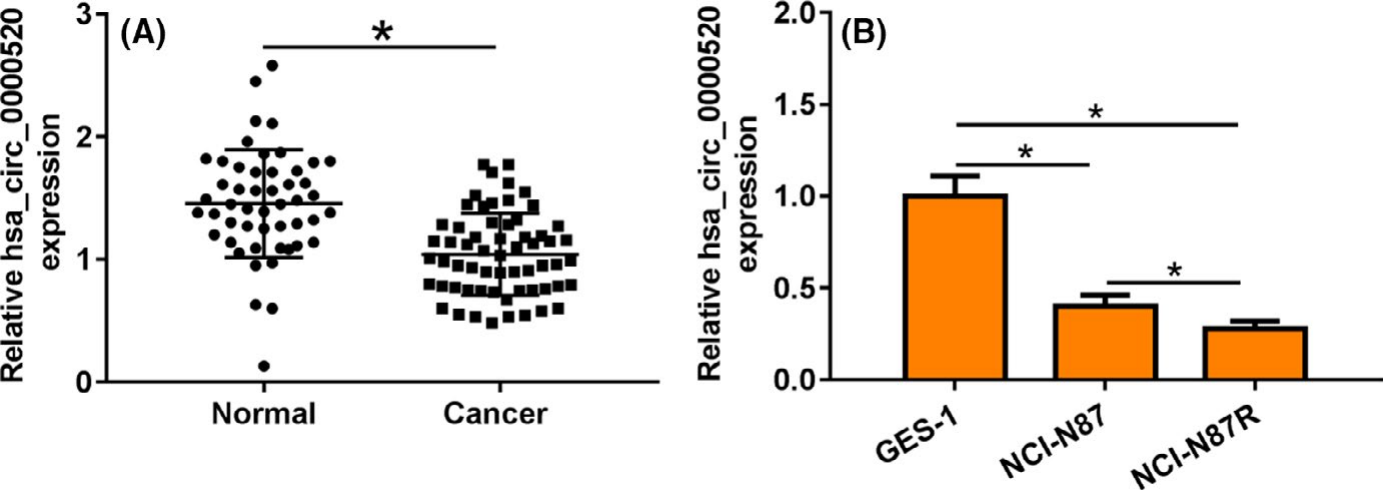
hsa_circ_0000520 expression in gastric cancer tissues and cells. A, The expression of hsa_circ_0000520 in gastric cancer tissues was detected by qRT‐PCR. B, The expression of hsa_circ_0000520 in gastric cancer cells was detected by qRT‐PCR *Note*: Compared with normal group, *P < .05; compared with GES‐1, *P < .05

We first collected cancer tissues and normal adjacent tissues from 30 gastric cancer patients and found that hsa_circ_0000520 was lowly expressed in cancer tissues (Figure 1A). In addition, hsa_circ_0000520 was decreased in NCI‐N87 cells than that in GES‐1 cells (Figure 1B). Moreover, we established gastric cancer cells NCI‐N87R of Herceptin‐resistant for 8 months, the MTT results showed that cell viability first decreased and then increased, and stabilized at 6 months (Figure S1), which indicated that Herceptin‐resistant cell NCI‐N87R was successfully established. Furthermore, we found hsa_circ_0000520 level was much lower upon Herceptin treatment (Figure 1B). Thus, hsa_circ_0000520 might be involved in the process of gastric cancer.

### 3.2 Overexpression of hsa_circ_0000520 reduced the resistance of NCI‐N87R to Herceptin

**FIGURE 2:**
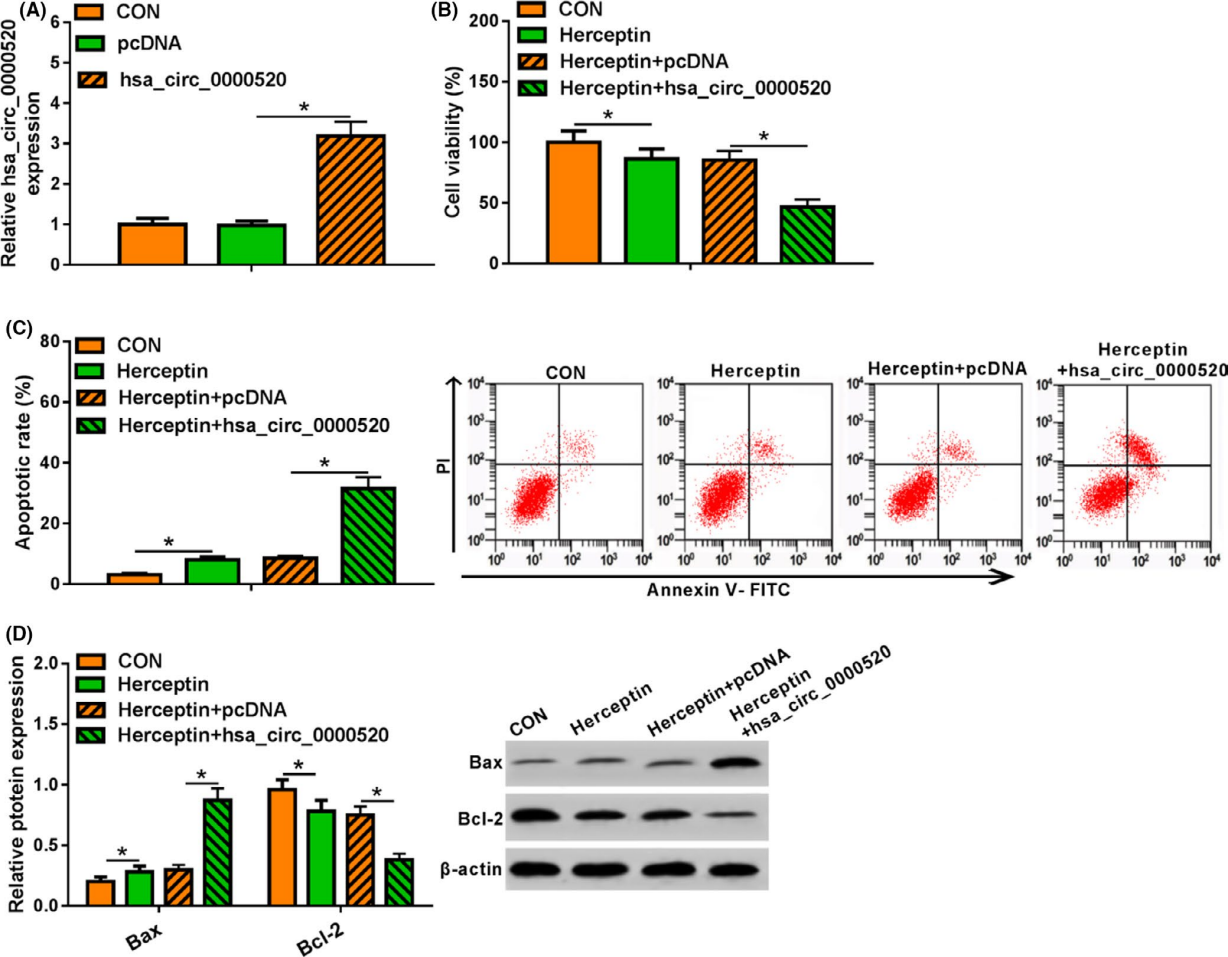
Overexpression of hsa_circ_0000520 decreased the resistance of gastric cancer cell line NCI‐N87R to Herceptin. A, The transfection effect of hsa_circ_0000520 was detected by qRT‐PCR. B, The effect of hsa_circ_0000520 overexpression on the proliferation of Herceptin‐treated NCI‐N87R cells was detected by MTT assay. C, The effect of hsa_circ_0000520 overexpression on apoptosis of Herceptin‐treated NCI‐N87R cells was detected by flow cytometry. D, The effect of hsa_circ_0000520 overexpression on the expression of apoptosis‐related proteins in Herceptin‐treated NCI‐N87R cells was detected by Western blot. *Note*: Compared with the CON (control) group, *P < .05; compared with the Herceptin + pcDNA group, *P < .05

To identify the role of hsa_circ_0000520 in Herceptin resistance of gastric cancer, we constructed hsa_circ_0000520 plasmid and transfected into NCI‐N87R cells. qRT‐PCR showed the transfected efficiency, and hsa_circ_0000520 was increased upon transfection (Figure 2A). Functional analysis showed that Herceptin treatment reduced cell viability, increased apoptosis rate, and promoted pro‐apoptosis–related protein expression, but inhibited anti‐apoptosis proteins level (Figure 2B‐D). However, hsa_circ_0000520 transfection further inhibited proliferation and promoted apoptosis in NCI‐N87R cells (Figure 2‐D). Together, hsa_circ_0000520 reduced Herceptin resistance in gastric cancer cell lines.

### 3.3 hsa_circ_0000520 inhibited PI3K‐Akt pathway in NCI‐N87R cells

**FIGURE 3:**
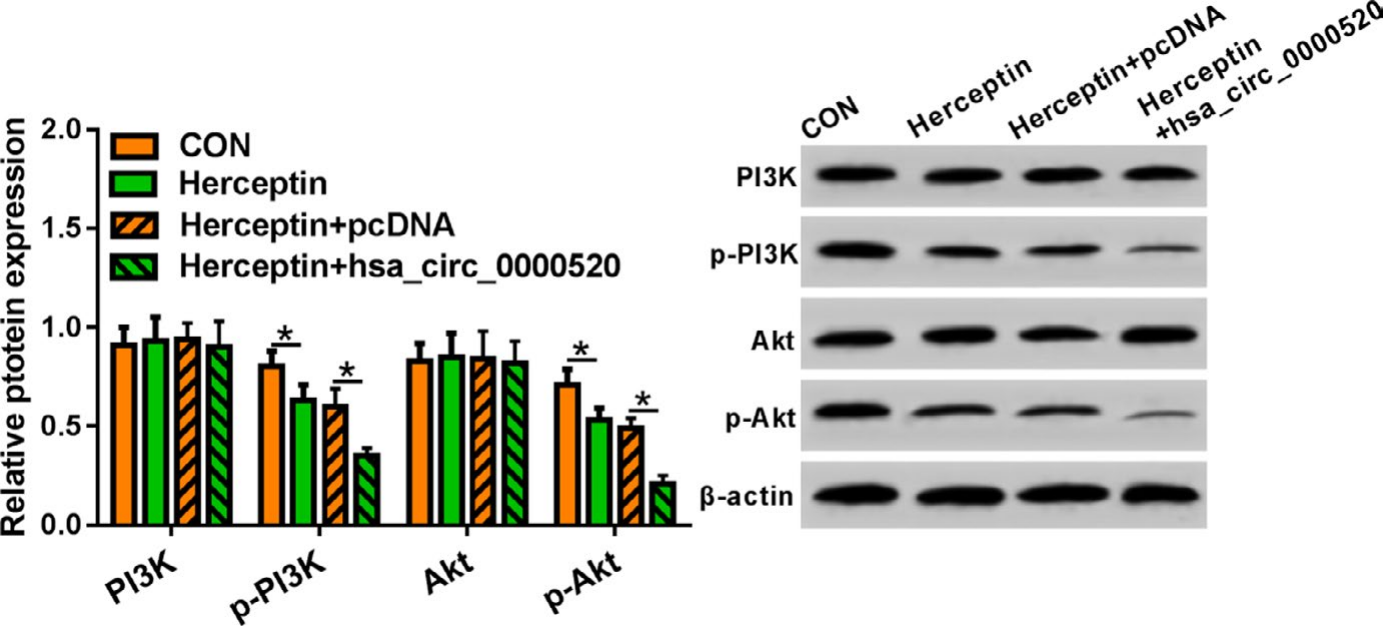
Effect of hsa_circ_0000520 overexpression on PI3K‐Akt pathway in NCI‐N87R cells. *Note*: Compared with the CON group, *P < .05; compared with the Herceptin + pcDNA group, *P < .05

Duo to the key function of PI3K/AKT in multiple cancers, we speculated that hsa_circ_0000520 might regulate PI3K/AKT in gastric cancer. To test our hypothesis, we forced expression of hsa_circ_0000520 in NCI‐N87R cells which suffered from Herceptin treatment. And p‐PI3K and p‐Akt were significantly reduced in Herceptin‐treated NCI‐N87R cells. Overexpression of hsa_circ_0000520 further inhibited p‐PI3K and p‐Akt expression (Figure 3). These data showed that hsa_circ_0000520 inhibited PI3K‐Akt pathway in gastric cancer.

### 3.4 hsa_circ_0000520 reduced the Herceptin resistance by deactivating PI3K‐Akt pathway in NCI‐N87R cells

**FIGURE 4:**
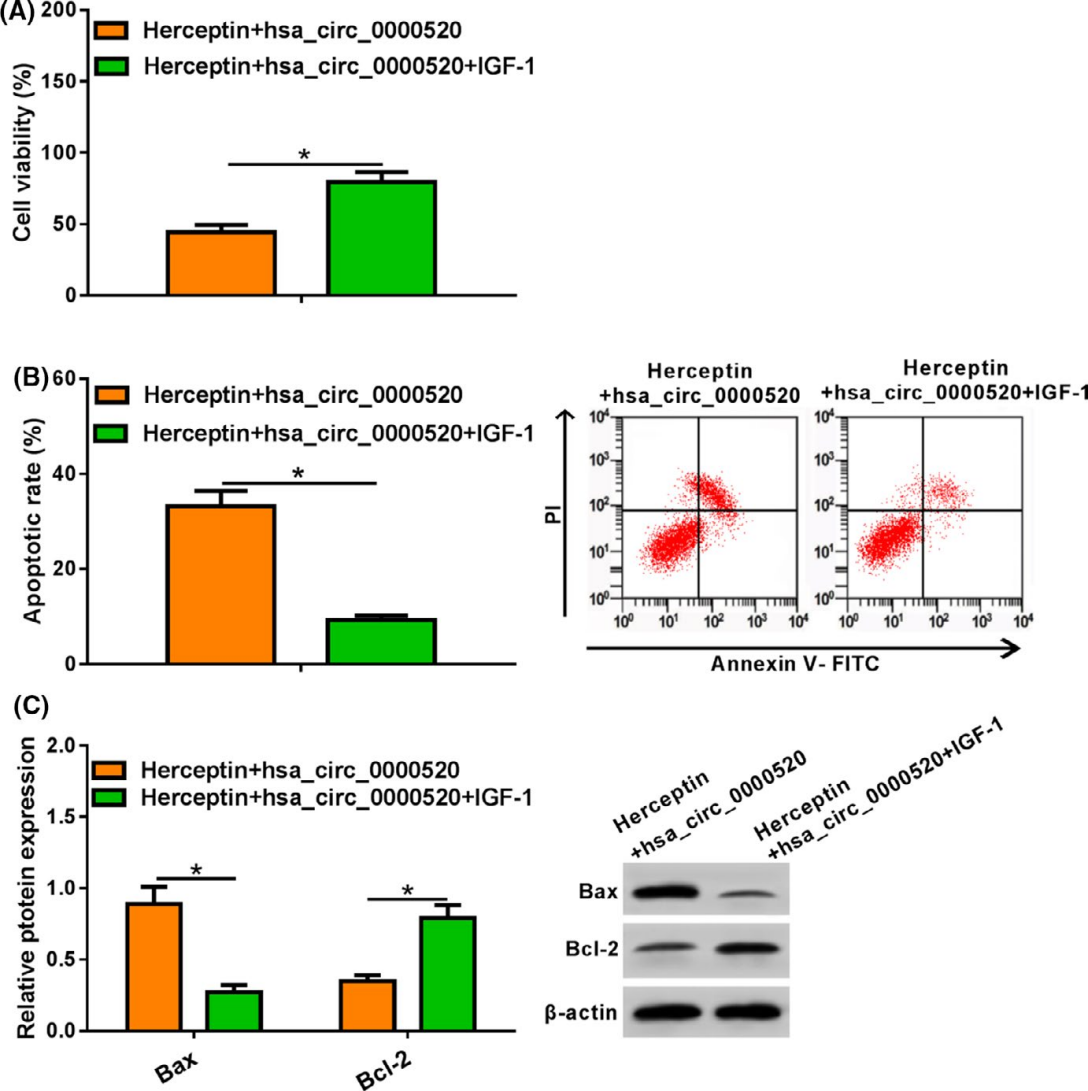
Effect of activating PI3K‐Akt signaling pathway on hsa_circ_0000520 overexpression and Herceptin‐treated NCI‐N87R cells. A. MTT was used to determine proliferation of NCI‐N87R cells. B, Apoptosis of Herceptin‐treated NCI‐N87R cells was detected by flow cytometry. C, Apoptosis‐related proteins was tested by Western blot. *Note*: Compared with Herceptin + hsa_circ_0000520 group, *P < .05

To further clarify the role of PI3K/AKT in hsa_circ_0000520 inhibition of Herceptin resistance in gastric cancer, we used IGF‐1 (an activator of PI3K/AKT) to treat NCI‐N87R cells with hsa_circ_0000520 transfection and Herceptin treatment. MTT assay showed that activating PI3K‐Akt pathway promoted cell viability (Figure 4A). In addition, flow cytometry and Western blot showed activating PI3K‐Akt pathway inhibited apoptosis of NCI‐N87R cells (Figure 4B and C). Taken together, activating PI3K‐Akt pathway removed the inhibitory effect of hsa_circ_0000520 on Herceptin resistance in gastric cancer.

## 4 DISCUSSION

As circRNA has the characteristics of stability and tissue specificity, thus it may become a tumor marker and be involved in progression of gastric cancer. Present study shows that Circ_0032821 promotes gastric cancer development by activating the MEK1/ ERK1/ 2; CircMRPS35 inhibits the progression of gastric cancer; Silencing RACRACGAP1 can enhance the sensitivity to Apatinib by targeting miR‐3657; CircRNA_0005075 inhibits gastric cancer growth via miR‐431/ p53/ epithelial stromal transformation axis; circ_0006282 promotes gastric cancer growth through miR‐155/ FBXO22 axis.^11^, ^12^, ^13^, ^14^, ^15^

However, it has not been elucidated about some mechanism of circRNAs in the process of gastric cancer. As it is reported that Herceptin can be used to treat gastric cancer, but patients are prone to Herceptin resistance, which limits the therapeutic effect.^16^, ^17^, ^18^ Therefore, it is of great significance to find the molecular mechanism and target genes for the treatment of Herceptin resistance.

Hsa_circ_0000520 may act as a tumor suppressor in gastric cancer and hepatocellular carcinoma, but the exact mechanisms are unclear.^19^, ^20^ Similar to the results of the above study, our study showed some results as follows: hsa_circ_0000520 was decreased in gastric cancer tissues and cells, and hsa_circ_0000520 in NCI‐N87R cells was significantly lower than that of NCI‐N87 cells. It indicated that hsa_circ_0000520 might contribute to the resistance of gastric cancer to Herceptin.

The proliferation of gastric cancer cells was significantly reduced after treatment with Herceptin, and hsa_circ_0000520 overexpression can significantly reduce the proliferation. Bcl‐2 is an anti‐apoptosis gene, while Bax promotes apoptosis.^21^, ^22^ Our study showed that Herceptin treatment promoted apoptosis, and the apoptosis rate increased significantly after hsa_circ_0000520 overexpression in gastric cancer cells. It is suggested that hsa_circ_0000520 reduced Herceptin resistance in gastric cancer cell lines.

It has reported that LncRNA AK023391 facilitates the occurrence and metastasis by activating the PI3K‐Akt signaling pathway.^23^, ^24^ Hsa_circ_0010882 also activates PI3K/ Akt signaling and promotes gastric cancer development.^25^ Previous studies p‐PI3K can activate Akt to form p‐Akt, and then participate in the process of gastric cancer development and development.^26^, ^27^, ^28^, ^29^, ^30^ It was shown p‐PI3K and p‐Akt were reduced after Herceptin treatment in gastric cancer cells, and hsa_circ_0000520 overexpression could restrain p‐PI3K and p‐Akt level. It is suggested that hsa_circ_0000520 may affect Herceptin resistance of gastric cancer through PI3K‐Akt pathway. In order to investigate whether hsa_circ_0000520 can play a role by regulating the PI3K‐Akt signaling pathway, the study will treat gastric cancer cells with PI3K‐Akt signaling pathway activator IGF‐1. As the results showed that cell viability was significantly increased and apoptosis was significantly reduced, Bax expression was down‐regulated, and Bcl‐2 expression was up‐regulated. It is suggested that hsa_circ_0000520 reduces Herceptin resistance via inhibiting the PI3K‐Akt in gastric cancer.

At present, the role and mechanism of ncRNAs are gradually clarified. Differential expression of ncRNAs may act as potential marker in differentiating gastric cancer types, stages, and metastases. With the development of molecular biology, people will have a deeper understanding of ncRNAs, which will benefit the diagnosis, treatment, and prognosis of gastric cancer.

In summary, hsa_circ_0000520 inhibited Herceptin resistance through PI3K‐Akt pathway in gastric cancer, which might provide a potential target for improving the diagnosis and treatment of gastric cancer.

## CONFLICT OF INTEREST

The authors declare no conflict of interest.

## Supporting information

Fig S1
