# Supplementary figures and images for: hsa_circ_0000520 influences herceptin resistance in gastric cancer cells through PI3K‐Akt signaling pathway
Source: J Clin Lab Anal. 2020 Jul 23;34(10):e23449. doi: 10.1002/jcla.23449 (PMC7595902; doi:10.1002/jcla.23449)

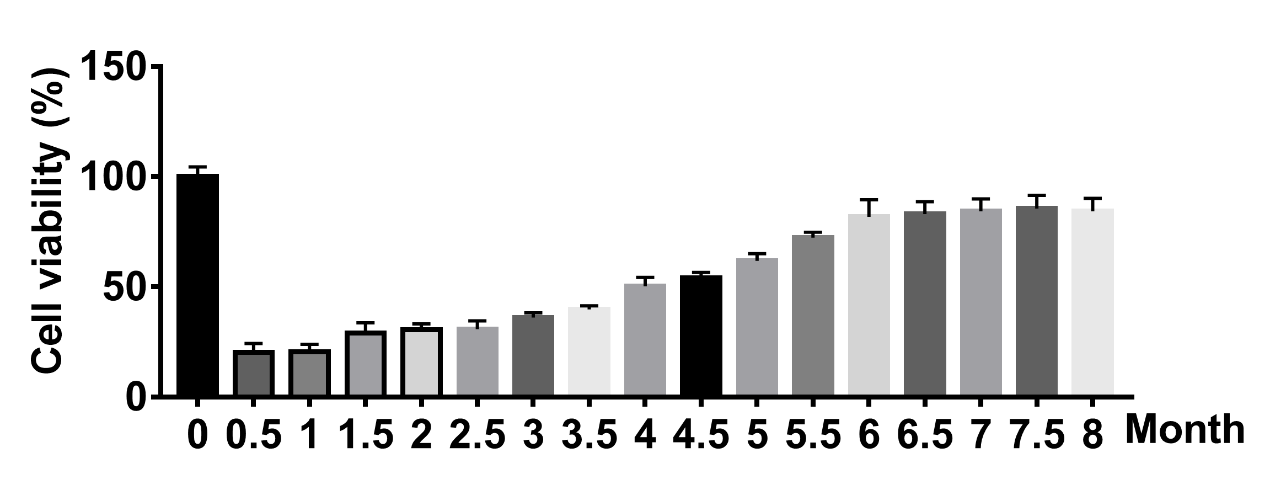

Supplement: Supplementary file 1 — Fig S1 [file JCLA-34-e23449-s001.tif]
